# High ocular CMV copies and mismatched receipts may predict poor visual prognosis in CMV retinitis patients following allogeneic haematopoietic stem cell transplantation

**DOI:** 10.1186/s12886-017-0622-0

**Published:** 2017-11-29

**Authors:** Yuehong Zhang, Xiangcai Ruan, Weizhong Yang, Ling Li, Zhuanhua Xian, Qiting Feng, Wenjian Mo

**Affiliations:** 1 Department of Ophthalmology, Guangzhou First People’s Hospital, Guangzhou Medical University, Guangzhou, 510080 China; 2 Departments of Anesthesia and Pain Medicine, Guangzhou First People’s Hospital, Guangzhou Medical University, Guangzhou, 510080 China; 3 Department of Medical Information, Guangzhou First People’s Hospital, Guangzhou Medical University, Guangzhou, 510080 China; 4 Department of Hematology, Guangzhou First People’s Hospital, Guangzhou Medical University, No.1 Panfu Road, Yuexiu District, Guangzhou, 510080 China

**Keywords:** Retina, Infection, Vision

## Abstract

**Background:**

To summarize the clinical characteristics and potential factors affecting the visual outcomes in patients with cytomegalovirus retinitis following allogeneic haematopoietic stem cell transplantation (HSCT).

**Methods:**

This retrospective study enrolled 12 patients (19 eyes) with cytomegalovirus retinitis after HSCT at Guangzhou First People’s Hospital in China between January 2013 and December 2014. Demographic and clinical characteristics, ocular manifestations and visual outcomes were evaluated by reviewing medical records at the Departments of Hematology and Ophthalmology. All patients were followed up at least 6 months after stopping antiviral therapy. The visual outcome was defined as improvement, stabilization and deterioration.

**Results:**

The subjects were composed of 7 human leucocyte antigen-matched and 5 mismatched receipts. All patients received combined systemic and intravitreous antiviral therapy. Eleven eyes gained improved or stabilized visual acuity, while 8 eyes suffered deterioration. Eyes with cytomegalovirus load less than 1 × 10^4^ copies/ml in vitreous accounted for higher rate in eyes with good visual prognosis than those with cytomegalovirus copies above 1 × 10^4^ copies/ml (52.63% vs 5.26%, *P* < 0.001). Human leucocyte antigen-matched receipts gained better visual prognosis than those mismatched ones (47.37% vs10.53%, *P* < 0.05). The virus types, cytomegalovirus peak in the blood, involved retinal zone and size had no influence on the visual outcomes (all *P* > 0.05).

**Conclusions:**

High ocular cytomegalovirus copies and mismatched receipts may be potential adverse factors affecting visual outcomes in cytomegalovirus retinitis patients following allogeneic HSCT.

## Background

Cytomegalovirus (CMV) retinitis is an ocular opportunistic infection in patients who fail to generate a T-cell response against CMV, which usually leads to a poor visual outcome due to its aggressive feature [1]. It typically afflicts immunocompromised patients including HIV infected people and patients with malignant diseases or any cases of receiving intensive immunosuppressive therapy [2–7]. CMV retinitis has been also reported in immunocompetent patients [8–10]. The diagnosis of CMV retinitis is usually made by a trained ophthalmologist by dilated fundus examination using indirect ophthalmoscopy. The ocular manifestation of CMV retinitis is variable due to different degree of CMV-specific leukocytes deficit and different severity of immunosuppression [11]. Most retinal lesions present irregular white necrotic foci or edema surrounded by granular infiltrates, spreading centrifugally along the vessels, with or without retinal hemorrhage. Some are mainly marked by retinal vasculopathy with vascular sheathing [12–14]. In some cases, keratic precipitates (KP), anterior chamber inflammation and vitritis may be also observed [2].

CMV retinitis after allogeneic haematopoietic stem cell transplantation (HSCT) has been concerned with the increment of HSCT recipients [6, 7]. HSCT is a well established treatment for many hematological diseases such as acute leukaemia, myelodysplastic syndromes and aplastic anemia. The donor for HSCT was initially limited to an identical twin, and then extended to human leucocyte antigen (HLA)-matched related or unrelated donor. Later, HSCT from a HLA-mismatched donor was also used in some patients lacking suitable HLA-matched donors [6]. The rapid progress of HSCT has significantly lowered the rates of transplant-related morbidity and mortality. However, the broadening of indication and the increase of transplant survivors after HSCT unfavorably increases many opportunistic infections including CMV retinitis [14, 15]. Here, we retrospectively described the clinical features of 12 CMV retinitis patients following allogeneic HSCT. The purpose of this study was to analyze the pattern of this disease and summarize the potential factors affecting visual prognosis in this specific population.

## Methods

We conducted a retrospective cohort study of CMV retinitis patients who received allogeneic HSCT at Guangzhou First People’s Hospital in China between January 2013 and December 2014. Collected data from an Electronic Patient Record included demographic data, clinical findings and virology record. Owing to the retrospective design and lack of complete follow-up for some patients, we excluded those who had been followed up less than 6 months after stopping antiviral therapy or whose data of ophthalmologic examination including fundus photograph and virus detection were incomplete. Patients who did not perform intravitreal injection of antiviral drug due to unstable condition after HSCT were also excluded. This study was approved by the institutional review board of Guangzhou First People’s Hospital and conducted in accordance with the Helsinki Declaration.

CMV retinitis was diagnosed by an experienced ophthalmologist using fundus pre-set lens with fully dilated pupils. All patients who received HSCT in our hospital were routinely monitored the viruses DNA levels including CMV and Epstein–Barr virus (EBV) in the peripheral venous blood using real-time, quantitative polymerase chain reaction analysis (PCR, the unit is copies/ml) before HSCT and then once weekly for at least three months. It was defined as CMV or EBV viremia when 2 consecutive levels of CMV-DNA > 500 copies/ml or EBV-DNA > 1000 copies/ml were detected in peripheral blood. When patients had gotten blurred vision or floaters scotoma or when they were suspected to suffer viral retinitis, they were referred to an ophthalmologist. The complete ophthalmologic examination at the initial visit and all follow-up visits included best-corrected visual acuity (BCVA), slit-lamp biomicroscopy, intraocular pressure measurement, and dilated fundus examination using fundus pre-set lens. We used a Snellen chart to assess visual acuity (VA) and the value was converted into1.0 LogMAR for statistical analysis. The VA of count fingers, hand movement, light perception and no light perception were recorded as 2.6, 2.7, 2.8 and 2.9 LogMAR units, respectively [2, 16]. The extent and size of retinal lesions were evaluated by fundus photographs. According to 3 distinct anatomical zones of the retina, zone 1 was defined in the area within 1500 μm of the optic nerve or within 3000 μm of the fovea, zone 2 was located from the edge of zone 1 anteriorly to the vortex veins and zone 3 was located anteriorly from zone 2 to the ora serrata. The sizes of lesions were classified as involving <10%, 10% to 50%, or >50% of the total retinal area based on the fundus photograph [17, 18].

All patients newly diagnosed with CMV retinitis undergo paracentesis of the vitreous to detect the DNA copies of CMV and EBV. Antiviral therapy included intravenous injection of ganciclovir (GCV, Cymevene, Roche Pharma Ltd., 5 mg/kg, q12h) or foscarnet solium (FOS, Jiangsu Chia-Tai Tianqing Pharmacy Co. Ltd, 60 mg/kg, q8h), and intravitreal injection of GCV (IVTG). The regimen of IVTG in our department is twice weekly for 2 weeks, 3 mg/0.1 ml, and maintains weekly injection with the same dose until the retinal lesions disappear or scar. All patients received IVTG with a 30-gauge needle 4 mm from the limbus under sterile condition. A 0.1 ml vitreous humor was sampled for DNA copies of virus just before the IVTG.

We assessed the antiviral therapy efficacy based on the final BCVA after stopping antiviral therapy. The visual outcome was defined as improvement, stabilization and deterioration. Improvement was defined if BCVA was increased two or more lines. Stabilization was defined if the changed BCVA was less than two lines. Deterioration was defined as a decreased BCVA more than two lines. In order to facilitate the statistical analysis, we defined the change of BVCA from light perception to hand movement, from hand movement to counting fingers as 2 line change in LogMAR vision [13].

Continuous data were presented as mean and standard deviation, whereas categorical data were presented as the number of suffered eyes and percentage. Analysis was performed for the influence of donor transplant, virus types in blood, CMV peak in the blood, CMV copies in the vitreous, involved retinal zone and size of lesions on the visual outcomes using Fisher’s Exact Test. We use Statistical Package for the Social Sciences, version 16 (SPSS Inc., Chicago, IL, USA) to analyze these data. *P* value less than 0.05 was considered to be statistically significant.

## Results

Twelve CMV retinitis patients (19 eyes) were ultimately included in this study. Primary hematological disorders treated with allogenieic HSCT included severe aplastic anemia (ten patients), acute myelocytic leukemia (one patient) and acute hybrid leukemia (one patient). The mean age of patients at presentation was 26.6 ± 9.0 years (range, 18–49 years) and 9 of them were male (75.0%).The donors of allogeneic HSCT included 7 HLA matched (3 related and 4 unrelated) and 5 HLA-mismatched donors. CMV viremia following HSCT was found in all patients and 6 (50%) suffered simultaneously EBV viremia before the diagnosis of CMV retinitis. The mean time of CMV viremia after HSCT was 29.67 ± 9.25 days, and time of CMV retinitis was 126.33 ± 46.52 days. DNA levels for CMV and EBV in vitreous showed that 15 eyes (78.95%) had positive CMV and 4 eyes (21.05%) were undetectable for any one of two viruses. At time of CMV retinitis diagnosis, patients were administered combined IVTG and systemic antiviral therapy.

**Fig. 1:**
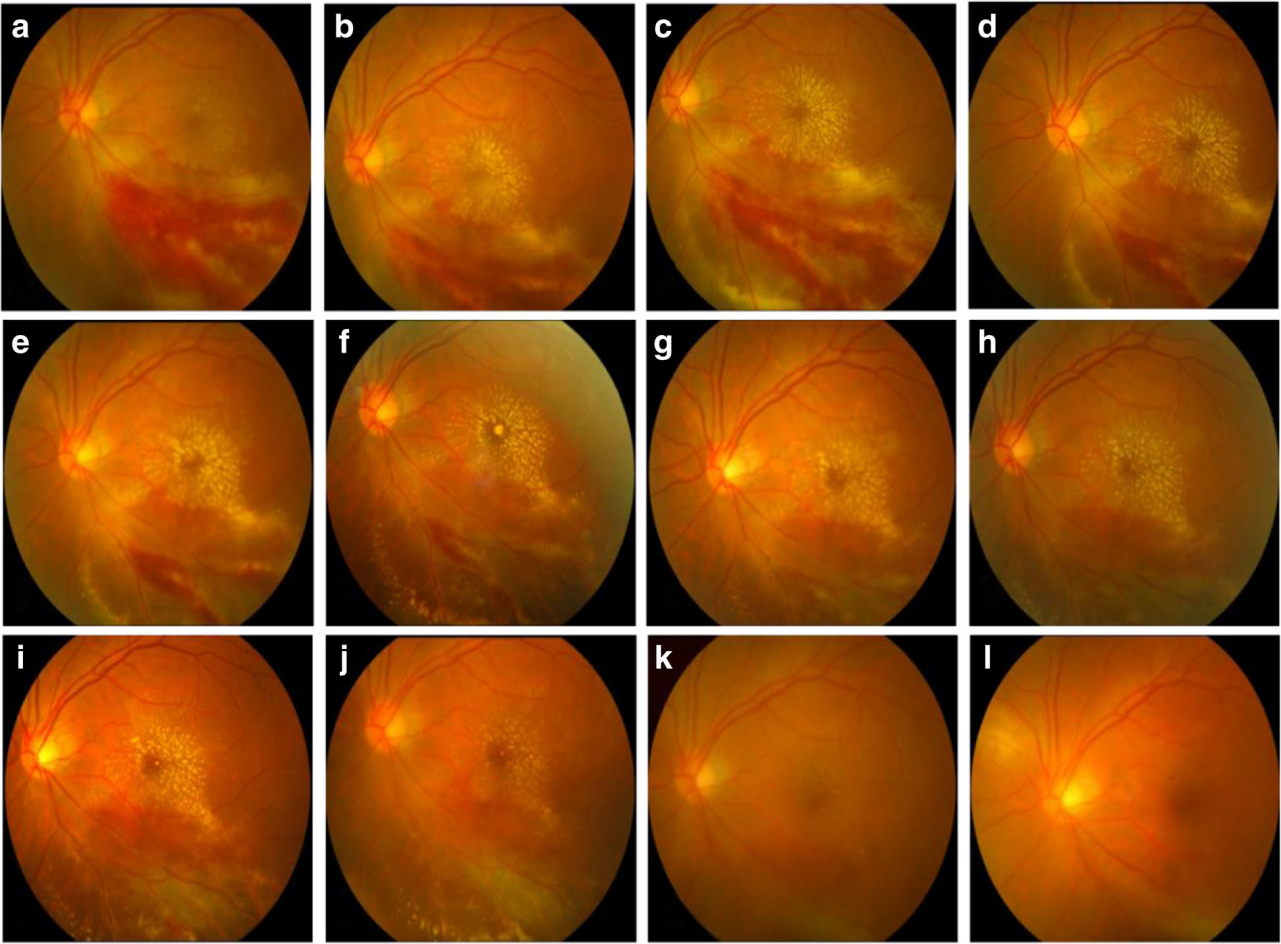
Fundus photography of the left eye showing the complete resolution of active retinitis after intravitreal injection of ganciclovir. **a** Left eye fundus photography showing active cytomegalovirus retinitis lesions at presentation. There is macular involvement. Initial visual acuity was 0.3 LogMAR. **b** Fundus photograph at the 3rd day after the first intravitreal ganciclovir injection in the left eye. **c** Fundus photograph at the 7th day after the second intravitreal ganciclovir injection in the left eye. **d**-**j** Fundus photography showing the gradually resolution of active lesions, with the remission of retinitis after treatment of weekly intravitreal ganciclovir injection. **k** Fundus photographs showing total resolution of active lesions at the 2nd month after the last intravitreal ganciclovir injection. **l** The fundus photography at the 5th month follow-up. Final visual acuity was 0.1 LogMAR

Table 1 demonstrated the ocular manifestations and treatment outcomes of these 12 patients. Five patients suffered unilateral CMV retinitis and 7 had bilateral involvement. When they first visited the ophthalmologist, KP was present in 4 eyes (21.1%), anterior chamber inflammation was present in 5 eyes (26.3%) and vitritis was present in 10 eyes (52.6%). Retinal lesions involving 3 zones and >50% of the total retinal area were found in 7 affected eyes (36.8%). Eyes presented with KP and aqueous flares were treated with Tobramycin and Dexamethasone Eye Drops (Alcon), and signs disappeared soon. Three eyes suffered relapse and the relapse time was 27 (case 1), 82 (case 4, OD) and 56 (case 5) days after combined IVTG and intravenous injection of GCV, respectively. Repeated combined antiviral therapy was administrated on these three eyes and two eyes received improved visual outcome (case 1 and 5) and one eye suffered deteriorated visual outcome (case 4). Figure 1 was exampled to present the process of lesion resolution after IVTG and simultaneously intravenous injection of GCV.

**Table 1:** Ocular manifestations and outcomes of antiviral therapy of 12 patients with CMV retinitis

No.	Involved eye	Presenting VA (LogMAR)	Involved zone	Sizes of lesions	KP	Anterior chamber inflammation	Vitritis	CMV peak in the blood	CMV copies in vitreous	No. of IVTG	Final VA (LogMAR)	Outcomes
1	OD	+0.1	3	<10%	Yes	Yes	Yes	7.3 × 10^3^	undetectable	8	0.0	Improvement
	OS	+0.1	3	<10%	No	Yes	Yes		undetectable	17	0.0	Improvement
2	OD	+2.6	1,2,3	>50%	No	No	No	1.9 × 10^5^	7.23 × 10^4^	13	+1.0	Stabilization
	OS	+2.6	1,2,3	>50%	No	No	No		8.65 × 10^3^	13	+1.0	Improvement
3	OD	+0.2	3	<10%	No	No	No	2.78 × 10^6^	undetectable	2	+0.1	Stabilization
4	OD	+0.2	1,2,3	>50%	No	No	Yes	2.4 × 10^4^	1.00 × 10^6^	25	+1.0	Deterioration
	OS	+0.1	1,2	10%–50%	No	No	Yes		2.34 × 10^5^	13	+0.4	Deterioration
5	OD	+0.1	1	<10%	No	No	No	1.0 × 10^4^	5.52 × 10^2^	7	−0.1	Improvement
	OS	+0.1	2	<10%	Yes	Yes	Yes		5.63 × 10^2^	21	0.0	Improvement
6	OD	0.0	3	<10%	No	No	Yes	4.4 × 10^3^	undetectable	4	0.0	Stabilization
7	OS	0.0	3	<10%	Yes	Yes	Yes	9.0 × 10^3^	5.34 × 10^2^	4	0.0	Stabilization
8	OD	+0.4	1,2,3	>50%	No	No	No		3.32 × 10^6^	28	+2.9	Deterioration
	OS	+0.5	1,2,3	>50%	No	No	No		3.68 × 10^6^	29	+2.9	Deterioration
9	OS	+0.3	1,2	10%–50%	No	No	No	3.5 × 10^3^	6.79 × 10^3^	9	+0.1	Improvement
10	OS	+0.2	2	10%–50%	No	No	No		6.24 × 10^2^	3	+0.2	Stabilization
11	OD	+0.1	3	<10%	No	No	No	4.4 × 10^4^	4.18 × 10^4^	8	+0.5	Deterioration
	OS	+0.2	2,3	10%–50%	Yes	Yes	Yes		1.85 × 10^5^	8	+2.6	Deterioration
12	OD	+1.0	1,2,3	>50%	No	No	Yes	3.3 × 10^3^	2.79 × 10^6^	17	+2.7	Deterioration
	OS	+0.2	1,2,3	>50%	No	No	Yes		2.24 × 10^6^	17	+2.6	Deterioration

VA: visual acuity; Presenting VA: vision acuity of the involved eye when CMV retinitis was first diagnosed; Final VA: vision acuity at the last visit; KP: keratic precipitates; OU: both eyes; OD: right eye; OS: left eye; involved zone of retinal lesions: zone 1 was in the area within 1500 μm of the optic nerve or within 3000 μm of the fovea, zone 2 was located from the edge of zone 1 anteriorly to the vortex veins and zone 3 was located anteriorly from zone 2 to the ora serrata. Sizes of lesions were classified as involving <10%, 10% to 50%, or >50% of the total retinal area based on the fundus photograph. Patient numbers correspond to those in Table 1

Table 2 showed the influence of donor transplant, virus types in vitreous and blood, CMV copies in vitreous, CMV peak in the blood, as well as involved retinal zone and size on the visual outcomes. Improved or stabilized visual outcomes were found in 11 eyes, while 8 eyes suffered deterioration. Of these eight eyes suffered visual deterioration, seven had optic atrophy and closure of the retinal vessels (both eyes of case 4, 8 and 12, and left eye of case 11), and one had rhegmatogenous retinal detachment (right eye of case 11). The right eye of case 11 underwent pars plana vitrectomy immediately after the diagnosis of rhegmatogenous retinal detachment. Eyes with CMV copies less than 1 × 10^4^/ml in vitreous accounted for higher rate in improved or stabilized eyes than those with CMV copies more than 1 × 10^4^/ml (*P* < 0.001). Patients who received HSCT from HLA-mismatched donor exhibited poorer prognosis than those who had fully matched donors (*P* < 0.05). Subanalysis also showed no significant difference in the influence of virus types, CMV peak in the blood, involved retinal zone and size on the visual outcomes (all *P* > 0.05).

**Table 2:** Subanalysis showing the influence of donor transplant, virus types and copies, involved retinal zone and size on the visual outcomes

Subanalysis	Eyes of improvement or stabilization, n (%)	Eyes of deterioration, n (%)	*P*
Donor HLA matching			
*Matched*	9 (47.37%)	2 (10.53%)	
*Mismatched*	2 (10.53%)	6 (31.58%)	0.022
Positive virus in blood			
*CMV + EBV*	6 (31.58%)	4 (21.05%)	
*CMV*	5 (26.32%)	4 (21.05%)	1.000
CMV or EBV virus in vitreous			
*Positive*	7 (36.84%)	4 (21.05%)	
*Negative*	4 (21.05%)	4 (21.05%)	0.103
CMV peak in the blood			
*CMV copies/ml >* 1 × 10^4^	6 (31.58%)	6 (31.58%)	
*CMV copies/ml <* 1 × 10^4^	5 (26.32%)	2 (10.53%)	0.633
CMV copies in vitreous			
*CMV copies/ml in vitreous >* 1 × 10^4^	1 (5.26%)	8 (42.11%)	
*CMV copies/ml in vitreous <* 1 × 10^4^	10 (52.63%)	0 (0)	0.000
Involved zone			
*Involved 3 zones*	2 (10.53%)	5 (26.32%)	
*Involved 1 or 2 zones*	9 (47.37%)	3 (15.78%)	0.074
Sizes of lesions			
*Sizes of lesions > 50%*	2 (10.53%)	5 (26.32%)	
*Sizes of lesions < 50%*	9 (47.37%)	3 (15.78%)	0.074

## Discussion

In this study, data was retrospectively analyzed to summarize the clinical and ocular manifestations as well as the antiviral therapy outcomes in 12 patients (19 eyes) with CMV retinitis following allogeneic HSCT. We found that 11 eyes obtained improvement or stabilization and 8 eyes suffered deterioration in visual outcomes following the antiviral therapy. All affected eyes with poor prognosis had CMV copies more than 1 × 10^4^/ml in vitreous. The 8 eyes with poor visual prognosis involved 4 patients 3of whom received HSCT from HLA-mismatched donors. These findings indicate that the viral copies in intraocular fluid and type of donor may be the potential factors affecting visual outcomes in CMV retinitis patients following allogeneic HSCT. This is predictable that CMV retinitis patients with high CMV copies in intraocular fluid and HLA-mismatched receipts will experience greater challenges in antiviral therapy.

We detected virus copies in the blood and vitreous samples using real-time quantitative PCR. All patients had positive CMV and/or EBV copies in the blood while 78.95% eyes had positive CMV copies in the vitreous. The CMV DNA was undetectable in four eyes of three patients (both eyes of case 1, and right eyes of case 3 and 6), and our CMV detection rate of intraocular fluid was in line with those reported by Iu LP et al. [2] (88.9%) and Agarwal A et al. [19] (82%). We guess two reasons contribute to the negative PCR findings. One is that cutoff values of quantitative PCR are not definitely appropriate for clinical decision-making. In our institution, we establish 500 copies/ml as the cutoff value for positive CMV infection and detectable CMV load less than 500 copies/ml is regarded as negative. Another is that the diagnosis of CMV viremia following the HSCT is about 97 days earlier than the time of diagnosis of CMV retinitis and all patients had received systemic antiviral therapy due to CMV viremia or other CMV end-organ diseases. However, diagnosis of CMV retinitis does not require laboratory tests or eye tests. Indirect ophthalmoscopy of the retina with fully dilated pupil is the gold standard for diagnosis of CMV retinitis [20–22]. In the present study, the three patients were diagnosed with CMV retinitis by a skilled ophthalmologist using this reliable standard at day 86, 207 and 81, respectively, after their HSCT, and were thus included in our final analysis.

We also found that 5 patients (41.67%) whose onset times of CMV retinitis was during the first 100 days after HSCT, which is comparable to findings from Yoo YS et al. [23] which reported that 50% cases occurring CMV retinitis were during the first 100 days after transplantation. However, the difference is that the earliest CMV retinitis case in our study was found in 81 days after HSCT, but that of Yoo YS et al. [23] was 27 days. Apparently, the diagnosis time of CMV retinitis in our study was later than theirs. We cannot rule out one possibility that some patients who had already suffered CMV retinitis did not receive the retinal examination timely due to unstable condition after HSCT. The fact that the presenting VA in some patients (such as case 2, 8 and 12 in Table 2) was poor may support this possibility. This finding prompted the importance of routine screening for CMV retinitis in patients following HSCT.

Our retrospective study included 5 patients who received HSCT from HLA-mismatched donors. As a well-established treatment option for many hematological diseases, HSCT has significantly lowered the rates of transplant-related morbidity and mortality with the advances of transplantation techniques. However, the fact is that only partial patients can timely find HLA matched related or unrelated donors for HSCT. Due to HLA polymorphism, limited pool of potential donors, as well as other reasons, many patients have no choice in donor selection and some of them are even doomed incurable. Fortunately, the broadening of HSCT indication brings opportunity of survival and hope to these patients whose donors are only partially HLA-matched. Some studies have outlined the positive impact of HSCT from HLA-mismatched donors on overall survival, while also noted its high rate of complications [6]. In our study, 8 eyes were diagnosed as CMV retinitis in theses 5 HLA-mismatched receipts. From the prognosis of visual outcomes, 6 eyes suffered deteriorated visual outcomes and 2 (case 8) of whom had a final VA of no light perception. Considering the complexity of prognostic factors, we cannot blame the poor visual outcomes of these 6 eyes on HLA-mismatched receipts only, but our finding seems to imply a higher rate of deterioration in visual outcomes among CMV retinitis patients who underwent HSCT from HLA-mismatched donors.

We did not find the significant difference in the influence of CMV peak in the blood, involved retinal zone and size on the visual outcomes, although high CMV peak in the blood and wide retinal lesions may imply the aggressive feature of CMV infection and delayed treatment. It has been shown that macular involvement was significantly associated with poor visual outcomes in CMV retinitis patients [2]. Some cases with macular involvement in our study surprisingly obtained an ideal curative effect, as the example in Fig. 1. We did not incorporate macular involvement into the subanalysis, so we cannot jump to a conclusion that macular involvement was a poor or irrelevant prognostic factor.

The limitations of this study include its small scale and the inadequate immune assessment of the included patients. Only the CMV retinitis, a rare complication after allogeneic HSCT, was retrospectively analyzed. To minimize the bias of the main outcomes, we applied standard ACTG criteria of “confirmed CMV retinitis” [20]. Different results and explanations may be obtained in patients with adequate assessment of the immune functions affecting the antiviral therapy efficacy, which should be a way to the next step. Another limitation is that we did not confirm the reason for the relapse. Three eyes suffered relapse in our study after combined IVTG and intravenous injection of GCV. Further efforts can be focused on exploring risks factors that could predict relapse by larger prospective controlled trials.

## Conclusion

In summary, the present study shows that high ocular CMV copies and HLA-mismatched receipts may be potential adverse factors affecting visual outcomes in CMV retinitis following allogeneic HSCT. This might have significant implications for routine screening and early diagnosis of CMV retinitis, especially in patients who received HSCT from HLA-mismatched donors.

## Abbreviations

BCVA: Best-corrected visual acuity
CMV: Cytomegalovirus
EBV: Epstein–Barr virus
FOS: Foscarnet solium
GCV: Ganciclovir
HLA: Human leucocyte antigen
HSCT: Haematopoietic stem cell transplantation
IVTG: Intravitreal injection of ganciclovir
kP: Keratic precipitates
PCR: Polymerase chain reaction analysis
VA: Visual acuity
